# Shashen-Maidong Decoction-Mediated IFN-*γ* and IL-4 on the Regulation of Th1/Th2 Imbalance in RP Rats

**DOI:** 10.1155/2019/6012473

**Published:** 2019-06-24

**Authors:** Yuan Yang, Yanping Zhou

**Affiliations:** ^1^Graduate School, Wuhan Sports University, Wuhan, China; ^2^Hubei University of Chinese Medicine, Wuhan, China

## Abstract

**Objective:**

Studying correlative changes of Th1/Th2 (Th, Helper T cells) related factor Interferon-*γ* (IFN-*γ*) and Interleukin-4 (IL-4) in the progression of radiation pneumonia (RP) rats and the efficacy of Shashen-Maidong decoction on these indexes to explore the immune mechanism of the decoction on the prevention and treatment of RP.

**Methods:**

Male 60 Sprague-Dawley (SD) rats were randomly divided into four groups. In addition to the normal control group taking saline, the other rats were set up RP model treated with Shashen-Maidong decoction or dexamethasone (DXM), respectively. The IFN-*γ* and IL-4 concentrations in serum and bronchoalveolar lavage fluid (BALF) of rats were tested in the 2^nd^ and 4^th^ week after radiation, and the relative ratio of IFN-*γ*/IL-4 was calculated.

**Results:**

(1) There was significant difference of serum IL-4 concentrations in group B (p<0.01) and extreme difference in groups C and D (p<0.001) compared with group A in 4^th^ week. Compared with group D, IL-4 concentrations in group B increased significantly in both 2^nd^ and 4^th^ week (p<0.01). Group B had significantly decreased IFN-*γ* concentrations in BALF (p<0.001) compared with group D in the 4^th^ week. And IFN-*γ* concentrations in BALF in group B were increased compared with group C in the 4^th^ week (p<0.05). (2) There was no difference of the relative ratio of IFN-*γ*/IL-4 at each time in groups B and A for both serum and BALF, while the ratios in groups C and D in 4th week in BALF were increased (p<0.05) compared to group A.

**Conclusion:**

Shashen-Maidong decoction can improve the immune function of RP rats by increasing IFN-*γ* concentration and decreasing IL-4 concentration, possibly by increasing the relative ratio of IFN-*γ*/IL-4 to regulate the immune imbalance of Th1/Th2.

## 1. Introduction

RP is an acute pulmonary-related disease with early injury of aseptic inflammation by chest radioactive radiation. The damage of collaterals in lung caused by radiation heat-toxicity developed from initial symptoms like occasional tachypnea and oppression in chest to parched mouth, cough, and even hemoptysis subsequently [1]. These symptoms are the lack of Qi (a generic concept, similar to “function”, especially for energy metabolism) and Yin (belonging to Yin-Yang theory; here is the implication of immunity) manifestations after the exogenous pathogenic caloradiance invades organisms. In addition, different degree of Primordial-Qi (the most essential Qi in body and the vital foundation of life, containing the ability of anti-immunity) can also reflect the strength of immune function. Organisms cannot resist exogenous pathogenic factors due to the deficiency of Fei-Qi (lung function) which may exacerbate the disease. It is suggested that the immune imbalance pathogenesis of RP can be elaborated and regulated by eliminating pathogen to support Qi and Yin, which can be used as the theoretical and experimental basis for clinical treatment.

Traditional Chinese Medicine (TCM) believes radioactive radiation, such as X-ray, has the characteristics of high heat-source property with fast speed and strong penetration. It can be classified as the pathogenic factor of heat-toxicity in Febrile disease which contains the pathogenic characteristics of external invasion and remarkable thermal property. This leads to the damage of Fei-Zang (lung and surrounding tissues) directly by depleting Qi and blood and finally deficiency of Yin. Shashen-Maidong decoction, the representative prescription of “Yang-Yin Sheng-Jin” method, nourishing Yin to promote the product of Jin (body fluid, whose function is similar to immunity), has good effects on the clinical treatment of early radiation-induced lung injury [2, 3]. We have carried on a long-term work on the mechanism of preventive and therapeutic efficacy on RP by Shashen-Maidong decoction [4–6]. This study furtherly explored the function of Shashen-Maidong decoction on the immune imbalance based on the “Yang-Yin Sheng-Jin” method.

**Figure 1:**
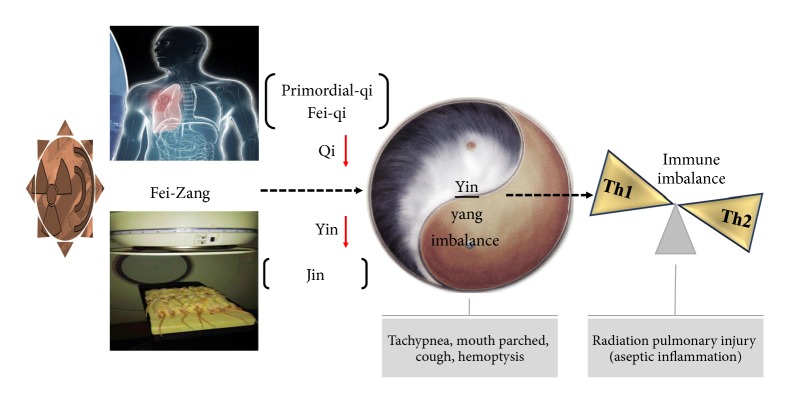
Relationship between radiation pneumonia and Yin-Yang theory.

At an earlier stage, there was a hot discussion on the relationship between TCM syndromes and immune cytokines, which induced the connection of Yin and immunity [7]. According to a study at present, the mechanism of RP relates to both immune imbalance and deficiency of Yin [8]. As shown in Figure 1, the disorder of proinflammatory factor and anti-inflammatory factor in the development of RP will lead to imbalance in cellular immunity, especially in the homeostasis of Th1/Th2. Besides, IFN-*γ* and IL-4 are the most typical cytokines of Th1 and Th2, respectively. Here is a hypothesis that Shashen-Maidong decoction can strengthen Yin and Jin to eliminate pathogenic factor to explore further mechanism of the prescription on the immune function of RP rats. Our research aims to provide a profound analysis for the application of Shashen-Maidong decoction in the treatment of RP. Namely, it can be highlighted by regulating Th1/Th2 and getting feedback on correlations through the concentration change and proportion of IFN-*γ* and IL-4 in the Lung and peripheral blood of RP rats.

## 2. Materials

### 2.1. Animal

Male 8-week-old SD rats were studied, which were bought from the Research Center of Laboratory Animal in Hubei Province, No. SCXK(e)20080005. These 60 rats weighing 220±50g were housed individually in stainless steel cages in a controlled environment (22±3°C, 40% ~ 70% humidity, with a 12-hour light and dark cycle) for 5-day acclimation period. All rats were given free diet and drinking water during the experiment. We ensured that all animals were normal so as not to affect the results of the experiment by keeping the lab clean, checking the equipment regularly, and observing the rats' diet, defecation, spirit, etc.

### 2.2. Prescription

Shashen-Maidong decoction (purchased from the Chinese Pharmacy of the first Hospital of Wuhan City) is composed of Coastal-Glehnia-root (Shashen) 18g, Phyllostachys officinalis (Hou Po) 12g, Ophiopogon japonicus (Maidong) 18g, Snake gourd root 9g, Lentils 9g, Mulberry leaf 9g, liquorice 6g, and wolfberry root-bar 18g. The original medicine preparation was placed in the rotary evaporator (Yarong, Shanghai). Then we obtained medicine solution with a concentration of 2g/ml which was concentrated at 90°C at 0.07mpa and stored at 4°C in a high pressure sterilization refrigerator.

### 2.3. Instruments and Reagents

6MV-X linear accelerator (Elekta, Sweden); FA2104 Electronic Analytical Balance (Shanghai Tianping, China); CS-15R centrifuge (Beckman, Germany); IFN-*γ* and IL-4 Elisa kit (Shenzhen Jingmei Biological Engineering, China); and enzyme-labeled tester (Labsystems, Finland) were used.

## 3. Methods

### 3.1. Establishment of RP Rat Model

Rats, weighed before irradiation, were anesthetized by 0.03ml/kg with 10% chloral hydrate. 6MV linear accelerator X-ray was used for single chest irradiation (total dose 20Gy). The model of RP rats was established in a relatively short time (see previous research protocol [5]). Ultraviolet radiation was used to sterilize the accelerator room before irradiation. This process was carried out in the Tumor Radiotherapy Department of the First Hospital of Wuhan City. After modeling, the rats were sent back to be kept in a routine way.

### 3.2. Grouping and Administration

60 rats were randomly divided into 4 groups: normal control group (group A), Shashen-Maidong decoction group (group B), dexamethasone treatment group (group C), and modeling control group (group D), with 15 rats each, respectively. Each rat was numbered and marked with picric acid, and then rats were grouped into cages. According to the “Methodology of Pharmacology of TCM” [9], the dosage by intragastric administration was calculated converting from the standard of adult physiological weight (the conversion coefficient of rat/200g corresponding to human/70kg was 0.018); we followed and calculated the dosage of Shashen-Maidong decoction as 5.15 ml/kg. Likewise, the dosage of DXM suspension was 5.2 ml/kg. Other groups (A, D) received the same amount of saline. Except group B, which began to take Shashen-Maidong decoction for 1 week before irradiation, other groups were given corresponding intragastric administration on the second day after modeling. The rats were weighed once a week during the intervention, and gastric perfusion was adjusted once a week according to the body weight change: weight gain = current week weigh — weight before experiment.

### 3.3. Specimen Collection

After 2 and 4 weeks of intragastric administration, 6 rats in each group were weighed and anesthetized with 10% chloral hydrate. Each rat toke 10 ml abdominal aorta blood for further testing. The lung tissues were isolated, and the left lung, 3ml × 3 times, was perfused with BALF. Then the serum (5000r/min,4°C, 5min) was taken from blood and lavage fluid after centrifugation and stored in -20°C refrigerator. The results were determined according to the instructions of rat IFN-*γ* and IL-4 Elisa kit.

### 3.4. Main Determination Methodology

*Histopathology Examination. * The lung tissues of rats were fixed with Formaldehyde, pruned and put into the embedding cassettes, dehydrated with alcohol, placed in xylene for transparency, and embedded with paraffin. Then we fixed them to the slicing machine and cut them into a 5 *μ*m thick slices. After that they were put into hot water to be smoothed, affixed to the slide, and dried in 45°C thermostat. Finally, we stained the slides after dewaxing and watering them.

*IFN-γ and IL-4 ELISA Kit Detection. *The kit was used to assay mouse IFN-*γ* and IL-4 level in the sample. Firstly, solid-phase antibody was made by purifying mouse IFN-*γ* and IL-4 antibody to coat microtiter plate wells. Then, IFN-*γ* and IL-4 were added to wells, and IFN-*γ* and IL-4 antibody were combined with HRP labeled to became antibody-antigen-enzyme-antibody complex. After complete washing, TMB substrate solution was added, which turned to blue color at HRP enzyme-catalyzed reaction. Finally, reaction was terminated by the addition of a sulphuric acid solution, and the color change was measured spectrophotometrically at a wavelength of 450 nm. The concentrations of IFN-*γ* and IL-4 in the samples were then determined by comparing the OD of the samples to the standard curve.

### 3.5. Statistical Analysis

The measured data were analyzed and processed by SPSS18.0, GraphPad Prism 5, Image-Pro Plus 6.0, and Curve Expert 1.3. T test and ANOVA test were used to analyze the significance of each index among different group samples. The data analyzed were expressed as Mean ± Standard Deviation (M±SD); p<0.05 indicates statistical difference, p < 0.01 indicates that each data item has significant difference, and p<0.001 indicates that each data item has extreme difference.

## 4. Results

### 4.1. Behavior Observation

Comparison between groups after irradiation: There was no obvious abnormality in group A. Within 2 weeks after irradiation, the rats in groups B, C, and D showed mild mental depression, decreased food intake, reduced mobility, slow response to stimuli, etc. Some rats even showed systemic hair loss and other symptoms such as perirhinal hemorrhage and periocular congestion. 2~4 weeks after irradiation, the spirit, diet, activity, smoothness of hair, and body weight of rats in group B were better than other modeling rats. Several rats in group C had shortness of breath apart from the symptoms before. The spirit of rats in group D was the worst; in addition to less activity and continued weight loss, the majority of these rats had eye-side and paranasal bleeding, increased periocular secretion, and dry stools.

### 4.2. HE Staining Observation

**Figure 2:**
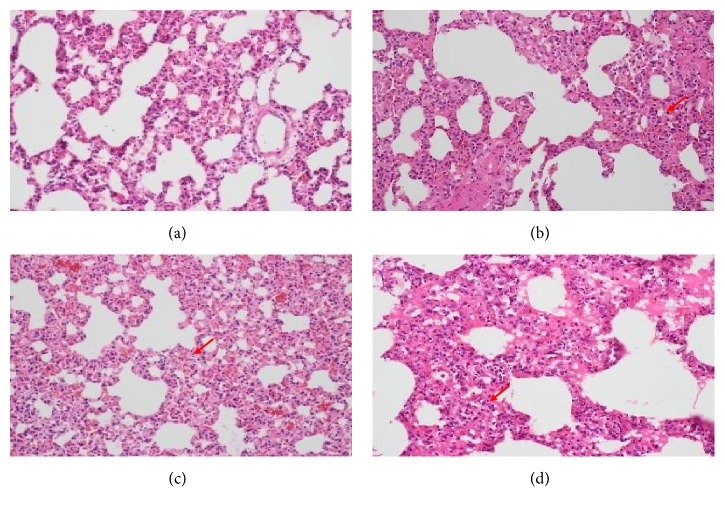
Pathological changes of lung in each group at the 4th week after gastric perfusion (HE staining, × 100).

The results of HE staining at 4 weeks after irradiation have shown more obvious difference than those at 2 weeks after irradiation in each modeling group, so we mainly focused on the specific analysis at 4-week cases. As Figure 2(a) shows, the structure of lung tissue was clear, and no obvious pathological changes were found in alveolar and alveolar septa in group A. In the other groups, varying degrees of inflammatory changes were observed mainly with macrophage and lymphocyte infiltration (marked with red arrow in the figure), accompanied by alveolar septa enlargement. Specifically, we can observe from Figure 2(d) that the alveolar septa in group D were slightly wider than others which showed that the interstitial edema was more obvious. The marked places show the inflammatory cells in groups B, C, and D such as mononuclear macrophages and lymphocytes in the alveolar septa and partial alveolar space. In group B and group C, the alveolar septa were enlarged and inflammatory cells were still infiltrated in part of alveolar cavity at the 4th week. However, the infiltration of inflammatory cells and the widening of alveolar septa in groups B and C were lighter than those in group D.

### 4.3. IFN-*γ* and IL-4 Concentrations in Serum and BALF

**Figure 3:**
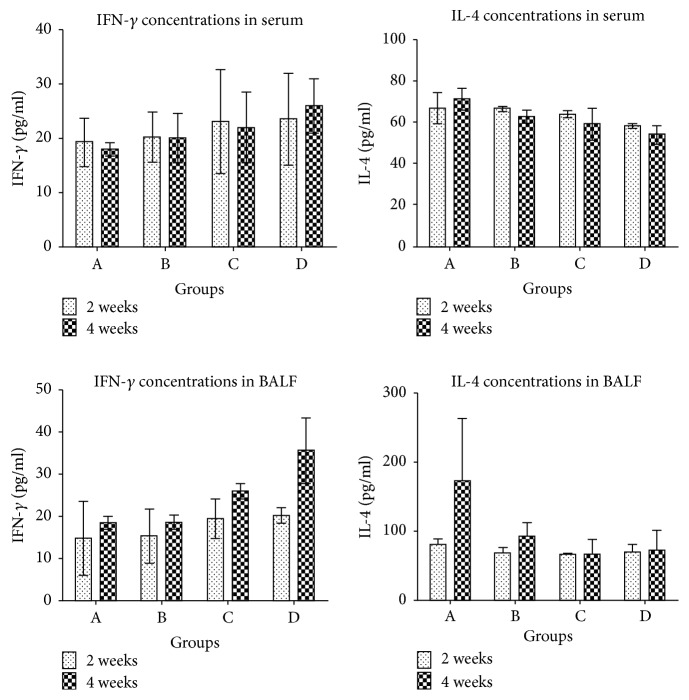
Changes of IFN-*γ* and IL-4 concentrations in serum and BALF.

Figure 3 shows the following: ① There was no statistical difference of IFN-*γ* concentrations in serum. ② IL-4 concentrations in serum: Compared with group A, only group D decreased significantly in 2 weeks (p<0.01); meanwhile, there was significant difference in group B (p<0.01) and extreme difference in groups C and D in 4 weeks (p<0.001). Compared with group D, group B increased significantly in both 2 weeks and 4 weeks (p<0.01). ③ IFN-*γ* concentrations in BALF: Compared with group A, group C increased in 4 weeks (p<0.05), and group D increased with extreme difference in 4 weeks (p<0.001). Compared with group D, group B decreased significantly in 4 weeks (p<0.001), and group C also decreased in 4 weeks (p<0.01). Compared with group C, group B increased in 4 weeks (p<0.05). ④ IL-4 concentrations in BALF: Compared with group A, the other three groups all decreased significantly in 4 weeks (p<0.001).

### 4.4. The Changes of IFN-*γ*/IL-4 Ratio

**Figure 4:**
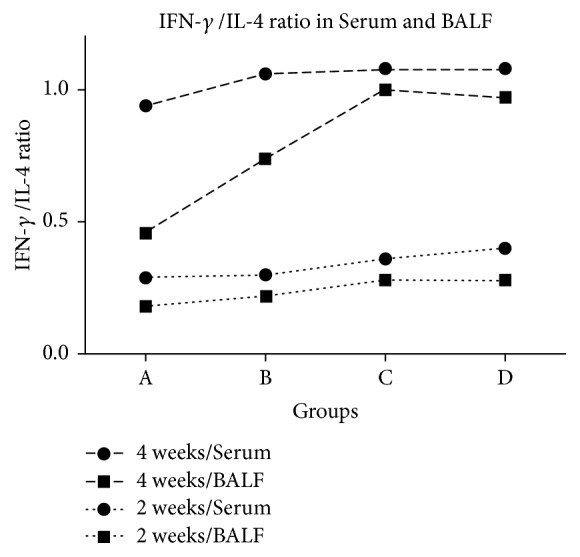
IFN-*γ*/IL-4 ratio in serum and BALF.

The ratio of IFN-*γ*/IL-4 was shown in Figure 4. Compared with group A, there was no difference at each time in group B for both serum and BALF, while the ratio in groups C and D at 4 weeks in BALF was increased (p<0.05).

## 5. Conclusions

### 5.1. Observation

The general situation of the rats after irradiation reflected that the modeling simulated the Yin deficiency syndrome of “heat-burn with Jin injury and deficiency of Qi and Yin”. We found that the spirit, diet, body weight, and hair removal of the rats with Shashen-Maidong decoction improved more obviously compared with the other modeling groups. Shashen-Maidong decoction could improve series of symptoms caused by heat-toxic injury and Yin deficiency of Fei-Zang, and our observation results also confirmed the obvious efficacy of this decoction in the treatment of RP.

The HE staining results showed that after 4 weeks of intragastric administration, the pathological inflammatory changes of lung tissues with Shashen-Maidong decoction intervention were significantly reduced compared with the model group, which meant Shashen-Maidong decoction could alleviate the inflammation of lung tissue in RP rats. In this experiment, however, the improvement of inflammation of this prescription was not significantly different from that of dexamethasone intervention. It is suggested that further researches can be done in the follow-up drug administration experiment with different length of time.

### 5.2. Analysis of IFN-*γ* and IL-4 in Serum and BALF

Based on the data above, we found that Shashen-Maidong decoction could significantly decrease the concentration of IFN-*γ*, increase the concentration of IL-4, and then regulate the ratio of IFN-*γ*/IL-4 in radiation modeling rats. This moderated anti-inflammation tendency after intervention seemed to be closer to the control nature rats. It also supported our hypothesis that Shashen-Maidong decoction possibly affected Th1/Th2 immune imbalance by regulating relative concentrations of IFN-*γ* and IL-4. And hence Shashen-Maidong decoction might inhibit the immune hyperactivity of Th1 and enhance the immune function of Th2 by changing related cytokines, thus regulating the imbalance of Th1/Th2 immunity and improving the immune function of RP rats. The results showed that the concentration of IFN-*γ* and IL4 in serum was slightly different from that in BALF which had more obvious changes. It was possible that the inflammation factors in blood did not adequately reflect the secretion and expression of the inflammatory cytokines because the more sensitive target tissue was Fei-Zang after radiation which might have more intense inflammatory response.

## 6. Discussion

Shashen-Maidong decoction, firstly shown in the book “Wen Bing Tiao Bian” [10] (a classic medical book which contained detailed analysis of epidemic warm diseases), was put forward by Wu Jutong in Qing Dynasty. This effective prescription aimed at the main treatment of “dryness-heat injury of Yin from Fei-Zang, being pyretic or causing cough”. The included eight herbs worked together to nourish the Yin and thus clear the effect of exogenous evil. In order to cope with the syndrome type of Yin deficiency, we analyzed these herbs that could specially act on the lung and related collaterals, such as Coastal-Glehnia-root, Phyllostachys officinalis, and Snake gourd root. Researches have shown that the main ingredients, such as Shashen polysaccharide [11], Maidong polysaccharide [12], Sangye polysaccharide [13], and Trichosanthin [14], could regulate the inflammatory environment after radiation. Meanwhile, Shashen-Maidong decoction was not greasy or cold with protecting digestion, and the sweet flavor could be easier to accept. As we mentioned this prescription had been recognized for its good effects which spread widely in clinics. Our experiment confirmed the function of its effects and extended discussion on the mechanism of treatment. Besides, this decoction had better target function on Fei-Zang superior to other organs which was in accordance with the specific sensitive messenger-induced function of these herbs in the past.

Th1 and Th2 are two subsets of Th cells, and the cytokines secreted by them can be regulated by cross-talking and maintain the normal immune function in vivo. Lung and surrounding tissues injury by radiation will directly induce inflammation, manifested by Th1 mediated hyperactivity of humoral immunity and relatively low cellular immunity mediated by Th2, finally resulting in RP immunity imbalance and pathological drift of Th1/Th2. The balance of Th1/Th2 refers to Yin-Yang theory in TCM. Specifically, RP is deficiency of Fei-Yin (Fei-Qi and Yin syndrome) and Yang syndrome is relatively hyperactive which corresponds to the imbalance of immunity. Shashen-Maidong decoction can nourish Fei-Yin and help to produce Jin, so that the cells and factors involved in immune response can maintain the balance of immunity and restore the steady internal environment. IFN-*γ* is a characteristic Th1 type cytokine, which can be used as an inflammatory mediator to promote the early symptoms of RP [15, 16]. IL-4 can promote the differentiation of Th2 cells and play an important role in enhancing cellular immunity and inhibiting humoral immunity [17, 18]. Thus, we can regulate the concentration of Th1 characteristic cytokine IFN-*γ* and Th2 related factor IL-4 so as to ameliorate the imbalance of Th1/Th2 immunity and alleviate the inflammatory injury of lung tissue [19, 20]. Our experiment has shown the regulation of Shashen-Maidong decoction to these two remarks which may be helpful to understand mechanisms that underlie maintenance of steady immunity and termination of aseptic inflammation.

Previous studies have showed that changes of INF-*γ* and IL-4 concentration and the relative changes of INF-*γ*/IL-4 reflect special herbal compound regulation on the balance of Th1/Th2 in many diseases [21–23]. Liang Fang specially focused on the imbalance of Th1/Th2 in lung cancer patients with deficiency of Qi and Yin to increase the immune function and then alleviate inflammation [24]. According to the clinical characteristic manifestations of RP and basis of the “Yin deficiency” theory combined with our early experimental results of Shashen-Maidong decoction, we continue the research to explore the therapeutic effect of supplementing Qi and nourishing Yin by regulating immune balance of Th1/Th2. This is a highlight of combining TCM method and evidence-based medicine (EBM) on RP and even some other similar condition of acute aseptic inflammation. In recent years, the mechanism of regulating cytokines in the treatment of RP has been involved; the conclusion of our experiment that Shashen-Maidong decoction regulates Th1/Th2 immune balance also reflects the progress from theoretical discussion to clinical research. We are working on the following work of exploring its mechanism based on recent researches such as immune homeostasis to provide better guide for clinical treatment. And for perspective of more related cytokines and other markers that can represent the immune function of Th1/Th2, we will focus on the specific targeted mechanism of Shashen-Maidong decoction on RP for further study.

## Data Availability

The data we used to support our findings of this study have been deposited in the CNKI database (details can be searched in the link: http://kns.cnki.net/kns/detail/detail.aspx?FileName=1016226374.nh&DbName=CMFD2017).
